# Heterogeneous hybrid immunity against Omicron variant JN.1 at 11 months following breakthrough infection

**DOI:** 10.1038/s41392-024-01898-x

**Published:** 2024-07-19

**Authors:** Xuan He, Jingyou Yu, Jiajing Jiang, Jinyuan Liu, Qi Qi, Dan Liu, Weimin Li

**Affiliations:** 1 Department of Pulmonary and Critical Care Medicine, Precision Medicine Key Laboratory of Sichuan Province, State Key Laboratory of Respiratory Health and Multimorbidity, West China Hospital, Sichuan University, Chengdu, Sichuan China; 2 Guangzhou National Laboratory, Bio-Island, Guangzhou, Guangdong China; 3 Sichuan Center for Disease Control and Prevention, Chengdu, Sichuan China

**Keywords:** Adaptive immunity, Infectious diseases

**Dear Editor**,

Due to the ongoing evolution of SARS-CoV-2 and waning immunity following COVID-19 vaccination, breakthrough infections have become common occurrences. The exposure to viral antigens during different COVID-19 waves and vaccination contribute to the unique “hybrid immunity”— immune protection conferred by the combined vaccination and natural infection in individuals.^1^ Recently, a highly transmissible SARS-CoV-2 variant JN.1, classified as a “variant of interest” by WHO, has become the predominant strain in China and worldwide. JN.1 significantly enhances its resistance to antibody response against SARS-CoV-2 by compensating the weakness of its predecessor BA.2.86 against class 1 neutralizing antibodies via the acquisition of an additional L455S.^2,3^ However, the current immunity against the circulating JN.1 at population level has yet to be fully evaluated.

To fill gaps in the understanding of immunity to the circulating JN.1, 139 individuals with heterologous combinations of COVID-19 vaccination and natural infection were recruited. The study primarily included mild cases of disease in vaccinated subjects with breakthrough infection (BTI) during the BA.5 wave, or with reinfection (RI) during the XBB/EG.5 wave (Fig. 1a). These COVID-19 convalescents were grouped by age and immunization history, and samples including blood and bronchoalveolar lavage (BAL) were collected at the indicated periods of time between May 2023 and January 2024 (Fig. 1a and Table S1).

**Fig. 1:**
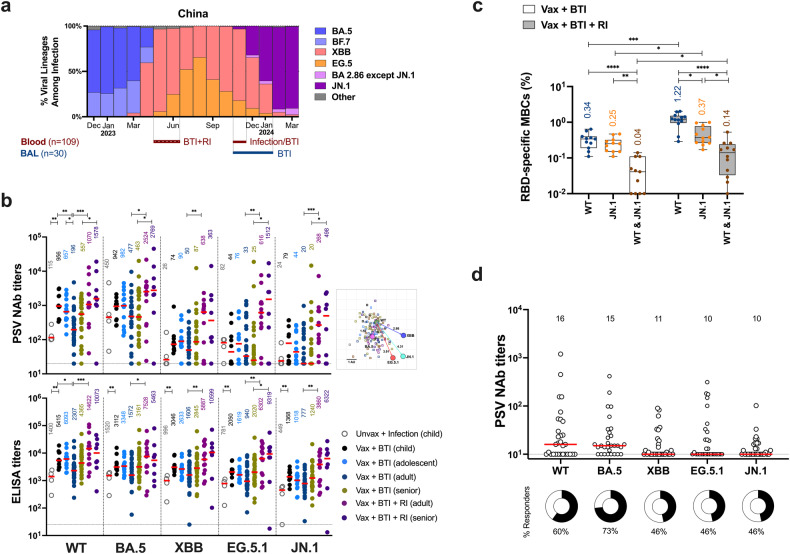
Humoral immunity against SARS-CoV-2 following BA.5-wave breakthrough infection. **a** Evolution of SARS-CoV-2 in China from December 2022 to March 2024. Data were collected from RCoV19 database of China National Center for Bioinformation. Blood (*n* = 109) and BAL (*n* = 30) samples were collected at the indicated periods of time. **b** Circulating antibodies against SARS-CoV-2 following BA.5-wave breakthrough infection (BTI). SARS-CoV-2 pseudovirus (PSV) neutralizing antibody (NAb) titers (above) and binding antibody titers (below) against wild-type (WT), BA.5, XBB, EG.5.1 and JN.1 were assessed in blood from subjects grouped as: unvaccinated (unvax) children with infection (*n* = 4); vaccinated (vax) children with BTI (*n* = 10); vaccinated adolescents with BTI (*n* = 14); vaccinated adults with BTI (*n* = 30); vaccinated seniors with BTI (*n* = 30); vaccinated adults with BTI plus RI (*n* = 13); vaccinated seniors with BTI plus RI (*n* = 8). The antigenic map was generated based on NAb data from all cohorts. The SARS-CoV-2 variants are shown as circles, and plasma samples are shown as empty squares. Both axes represent the antigenic unit (AU) corresponding to fold change in NAb titers. **c** Circulating memory B cells (MBCs) responding to WT and JN.1 in blood from BTI groups with RI (*n* = 12) or without RI (*n* = 11). **d** Respiratory mucosal antibody response against SARS-CoV-2 following BA.5-wave BTI. NAb titers against WT and Omicron lineages were assessed in bronchoalveolar lavage (BAL) from vaccinated subjects with BTI (*n* = 30). The proportion of responders (with NAb titers above the detection limit) is shown as the pies with numbers below. For all the graphs, bold red horizontal lines and numbers above reflect median values. Box and whisker plots reflect interquartile ranges (boxes), medians (horizontal lines), and range (whiskers). Dotted lines reflect lower limits of quantitation. Statistical analysis was performed using two-tailed Mann–Whitney (for two groups) or one-way ANOVA with a Kruskal–Wallis test (for more than two groups). *p < 0.05, **p < 0.01, ***p < 0.001, ****p < 0.0001

To determine the humoral immunity against SARS-CoV-2 in these subjects, we assessed the pseudovirus (PSV) neutralizing antibody (NAb) titers against the original wild-type (WT) strain and the primary Omicron variants including BA.5, XBB, EG.5.1 and JN.1 in blood. In general, the BTI without RI cohorts exhibited negligible NAbs against JN.1 with a median titer of 24.5 (interquartile range [IQR]: 20 to 122), while maintaining NAbs against WT and BA.5 with 17-fold and 25-fold higher than JN.1-specific NAbs, respectively, at 11 months post-BTI (*P* < 0.0001 for WT and BA.5) (Fig. 1b). We next evaluated the humoral immunity across groups of different ages following BA.5-wave BTI. Unlike unvaccinated children showing inferior NAb response, vaccinated children and adolescents preserved NAb responses against WT and variants, with 1.3 ~ 4.9-fold higher titers than vaccinated adults following BTI (*P* < 0.05 for WT) (Fig. 1b). Although the majority of subjects in the BTI without RI cohorts showed JN.1-specific NAb titers below 100, 80% (8/10) of children with a median titer of 78.5 (IQR: 22.3 to 227) and 78% (11/14) of adolescents with a median titer of 44 (IQR: 23 to 175.5) exhibited detectable NAb titers against JN.1, as opposed to less than half in the adults and seniors showing detectable JN.1-specific NAbs (Fig. 1b). Of note, reinfection during the XBB/EG.5 wave post-BTI led to 2.8 ~ 60.5-fold increase in NAb titers against WT and Omicron variants compared to cohorts without RI among adults and seniors (Fig. 1b). Particularly, elevated NAb response against the fastest-growing JN.1 was observed in BTI plus RI cohorts compared to those without RI, exhibiting a 13.4-fold increase in adults with a median titer of 268 (IQR: 84.5 to 695), and a 24.9-fold increase in seniors with a median titer of 498 (IQR: 23.75 to 1909) (Fig. 1b). In the antigenic map generated from the NAb data, BA.5 clustered together with WT (1.5 AU), while other variants were more antigenically distinct from WT (2.9 ~ 4.3 AU) with JN.1 being the furthest away, suggesting heightened antibody resistance of JN.1 relative to its predecessors (Fig. 1b). SARS-CoV-2 RBD IgG ELISA binding titers largely matched the NAb profile, showing significantly lower JN.1-specific IgG titers than that against WT and BA.5 in BTI cohorts (*P* < 0.05 for WT and BA.5), and reinfection post-BTI boosted the RBD IgG titers against WT and Omicron variants, including emerging JN.1, by 1.9 ~ 5-fold increase compared to that in cohorts without RI (Fig. 1b).

While achieving sterilizing immunity against SARS-CoV-2 requires adequate NAbs, protection against clinical disease can be attained through immune memory scenarios. Given the durable memory B cell (MBC) response against SARS-CoV-2,^4,5^ we examined circulating RBD-specific MBCs and observed detectable MBCs responding to WT and JN.1 in blood from BTI subjects with or without RI (Fig. 1c). Of note, XBB/EG.5-wave RI drove the expansion of MBCs responding to JN.1 (*P* < 0.05) and to both strains (*P* < 0.05), along with the increase of WT-specific MBCs (*P* < 0.001) (Fig. 1c). The immunological recall of MBCs responding to the ancestral viral strain following XBB/EG.5-wave RI suggested the involvement of immune imprinting.

Profiling the humoral immunity against SARS-CoV-2 in respiratory tract, the primary site of entry for coronavirus, is crucial. However, it is currently unclear whether efficient mucosal responses against Omicron lineages can be maintained over an extended period of months post-BTI. Here, we assessed mucosal NAb response against SARS-CoV-2 in BAL collected over 11 months following BA.5-wave BTI. More than 60% of individuals showed above-the-threshold yet low level of NAbs against WT and BA.5, while 46% of subjects showed detectable but marginal NAbs against XBB, EG.5.1 and JN.1 in BAL (Fig. 1d).

Overall, this study has provided critical evidence elucidating the current immunity against the newly emerging JN.1 at population level. Our findings reveal the heterogeneous hybrid immunity against SARS-CoV-2 over 11 months following breakthrough infection, and highlight the susceptibility of individuals, particularly high-risk seniors, to breakthrough infection by JN.1 in the ongoing COVID-19 landscape. An additional booster with XBB-containing vaccine may greatly alleviate the onward transmission of immune-evasive SARS-CoV-2 variants. The limited number of individuals per group in the study might hinder the generalizability of our findings. Further studies monitoring the population-level immunity against the evolving SARS-CoV-2 over time may remain necessary.

### Supplementary information

Heterogeneous hybrid immunity against Omicron variant JN.1 at 11 months following breakthrough infection

## Data Availability

Data in the manuscript are available on reasonable request.
