# Supplementary material for: Heterogeneous hybrid immunity against Omicron variant JN.1 at 11 months following breakthrough infection
Source: Signal Transduct Target Ther. 2024 Jul 19;9:180. doi: 10.1038/s41392-024-01898-x (PMC11258359; doi:10.1038/s41392-024-01898-x)
Supplement: Supplementary file 1 — Heterogeneous hybrid immunity against Omicron variant JN.1 at 11 months following breakthrough infection [file 41392_2024_1898_MOESM1_ESM.docx]

Supplementary Materials for

**Heterogeneous hybrid immunity against Omicron variant JN.1 at 11 months following breakthrough infection**

Xuan He^1,4*^, Jingyou Yu^2,4^, Jiajing Jiang^1,4^, Jinyuan Liu^1^, Qi Qi^3^, Dan Liu^1*^, Weimin Li^1*^

^1^Department of Pulmonary and Critical Care Medicine, Precision Medicine Key Laboratory of Sichuan Province, State Key Laboratory of Respiratory Health and Multimorbidity, West China Hospital, Sichuan University, Chengdu, Sichuan, China; ^2^Guangzhou National Laboratory, Bio-Island, Guangzhou, Guangdong, China; ^3^Sichuan Center for Disease Control and Prevention, Chengdu, Sichuan, China; ^4^ These authors contribute equally to the work.

Correspondence to: hexuan09@scu.edu.cn (X.H.); liudan10965@wchscu.cn (D.L.); weimi003@scu.edu.cn (W.M.L.)

**This PDF file includes:**

Materials and Methods

Tables S1

Materials and Methods

**Human cohorts**

The protocol for sample collection was approved by Ethics Committee Institution at Sichuan Center for Disease Control and Prevention and West China Hospital. We recruited the cohorts of 139 subjects between May 2023 to January 2024. In the cohorts, all vaccinated subjects were immunized with at least one dose of inactivated vaccines of either CoronaVac (Sinovac) or BBIBP-CorV (Sinopharm) at 0.5 ml per dose, and the last dose was received between August 2021 and February 2022. All COVID-19 cases in this study were confirmed using SARS-CoV-2 PCR, antigen test kits, or serodiagnostics, and predominantly comprised mild cases. The vaccinated cohort experienced breakthrough infection (BTI) during the BA.5 wave from December 2022 to February 2023, and samples including blood and bronchoalveolar lavage (BAL) were collected. For those BTI subjects experiencing reinfection during the XBB/EG.5 wave, blood samples were collected within two weeks post-symptom onset. Blood was collected in EDTA tube. For BAL collection, the bronchoscope was securely wedged into an airway, guiding towards a specific segment of the lung. About 20 ml of lavage fluid was collected. The fluid collected was placed on ice and transferred immediately to the laboratory for processing.

**Enzyme-linked immunosorbent assay**

The Enzyme-linked immunosorbent assay (ELISA) were conducted to measure the titers of binding antibody against the SARS-CoV-2 WT, XBB, BA.5, EG.5.1 and JN.1 (Sino Biological). ELISA plates (Corning) were coated with SARS-CoV-2 RBD protein at 1μg/mL in PBS overnight at 4 °C. The plates were then blocked using blocking buffer (PBST containing 2% BSA) at room temperature for 3 h. After washing with PBST buffer (MCE), human serum samples were serially diluted three-fold and added to the blocked plates, followed by a 1 h incubation at room temperature. Plates were washed with PBST three times. Subsequently, goat anti-human IgG antibodies (Invitrogen) were added and incubated for 1 h. Plates were washed once with PBST, and then added 100 μL per well of 3,3′,5,5′-tetramethyl biphenyl diamine (Life-iLab) and developed the plates for 10 min at room temperature in the dark. Finally, 50 μL of 1.0 M H_2_SO_4_ was added to each well, and measured the absorbance values at 450 nm on a microplate reader (Biotek).

**Production of pseudotyped lentiviral particles**

The SARS-CoV-2 pseudoviruses expressing a luciferase reporter gene were generated in an approach similar to as described previously. 10 µg packaging construct psPAX2, 10 µg luciferase reporter plasmid pLenti-CMV Puro-Luc, and 5 µg spike protein expressing pcDNA3.1-SARS CoV-2 SΔCT were co-transfected into 5 x 10^6^ HEK293T cells in T-75 flask with lipofectamine 2,000 (Sigma). Six hours post-transfection, the supernatants were replaced with fresh DMEM (plus 5% FBS). The supernatants containing the pseudotype viruses were collected 48 hours post-transfection; pseudotype viruses were purified by filtration with a 0.45 µm filter.

**Lentiviral luciferase-based neutralization assay**

To determine the neutralization activity of the plasma or BAL, HEK293T-hACE2 cells were seeded in 96-well tissue culture plates at a density of 2 × 10^4^cells/well overnight. Three-fold serial dilutions of heat-inactivated plasma samples or untreated BAL fluid were prepared and mixed with 50 µL of pseudoviruses. The mixture was incubated at 37 °C for 1 h before being added to HEK293T-hACE2 cells. Moreover, 48 h after infection, cells were lysed in firefly Luciferase Reporter Gene Assay Kits (Beyotime, RG006) according to the manufacturer’s instructions. SARS-CoV-2 neutralization titers were defined as the sample dilution at which a 50% reduction in the relative light unit (RLU) was observed relative to the average of the virus control wells.

**Antigenic cartography**

The Racmacs package (https://github.com/acorg/Racmacs, version 1.2.9) was used for antigenic cartography analyses. Antigenic map was constructed based on a modified multi-scaling approach to quantify and represent the relationship between serum antibody titer data and SARS-CoV-2 variants (antigens) in a two-dimensional space. The relative positions of each SARS-CoV-2 variant and sera sample were optimized. The spacing between grid lines on the maps is one antigenic unit (AU) corresponding to fold change in antibody titers.

**B cell immunophenotyping**

Human PBMCs were stained with Aqua live/dead dye (Invitrogen) for 20min at room temperature, washed twice with 2% FBS/PBS buffer, and incubated with Fc Block (Biolegend) for 10 min at room temperature. After blocking, samples were stained with anti-CD3 (BD Biosciences, clone UCHT1, PerCP-Cy5.5, 1:200), anti-CD14 (BD Biosciences, clone M5E2, PerCP-Cy5.5, 1:50), anti-CD16 (BD Biosciences, clone 3G8, PerCP-Cy5.5, 1:100), anti-CD56 (BD Biosciences, clone B159, PerCP-Cy5.5, 1:200), anti-CD27 (BD Biosciences, clone M-T271, PE-CF594, 1:200), anti-CD38 (BD Biosciences, clone HIT2, PE-Cy7, 1:200), anti-CD21 (BD Biosciences, clone B-ly4, brilliant violet (BV) 711, 1:100), anti-CD11c (BD Biosciences, clone B-ly6, Alexa Fluor 700, 1:100), anti-CD19 (BD Biosciences, clone SJ25C1, BUV395, 1:50), anti-CD71 (BD Biosciences, clone L01.1, BUV737, 1:100), anti-IgD (BD Biosciences, clone IA6-2, APC-H7, 1:50), biotinylated SARS-CoV-2 (JN.1) spike RBD protein (Sino Biological), SARS-CoV-2 (JN.1) spike RBD protein (Sino Biological) labeled with DyLight 405, SARS-CoV-2 (2019-nCoV) spike RBD protein (Sino Biological) labeled with fluorescein isothiocyanate (FITC) and allophycocyanin (APC) for 30min at 4℃. Subsequently, cells were washed twice with 2% FBS/PBS buffer, followed by incubation with BV650 streptavidin (BD Pharmingen) for 10 min at room temperature, then washed twice with 2% FBS/PBS buffer and fixed with 2% paraformaldehyde. All data were acquired on BD Fortessa flow cytometer. Subsequent analyses were performed using FlowJo software (BD Bioscience, v.10.8.1). For analyses, in singlet gate, dead cells were excluded by LIVE/DEAD Fixable Aqua Dead Cell Staining, and B cells were identified as CD19+CD3-CD14-CD16-CD56-. SARS-CoV-2 WT or JN.1 RBD-specific B cells were identified as double-positive for RBD proteins labeled with different fluorescent probes.

**Statistical analysis**

Statistical analyses of immunologic data from the study were performed using GraphPad Prism 9 (GraphPad Software). Comparisons of data between groups were performed using two-tailed Mann-Whitney (for two groups) or one-way ANOVA with a Kruskal–Wallis test (for more than two groups). P values of less than 0.05 were considered significant.

Table S1.

The demographic characteristics of grouped convalescents donating blood or bronchoalveolar lavage (BAL)

**
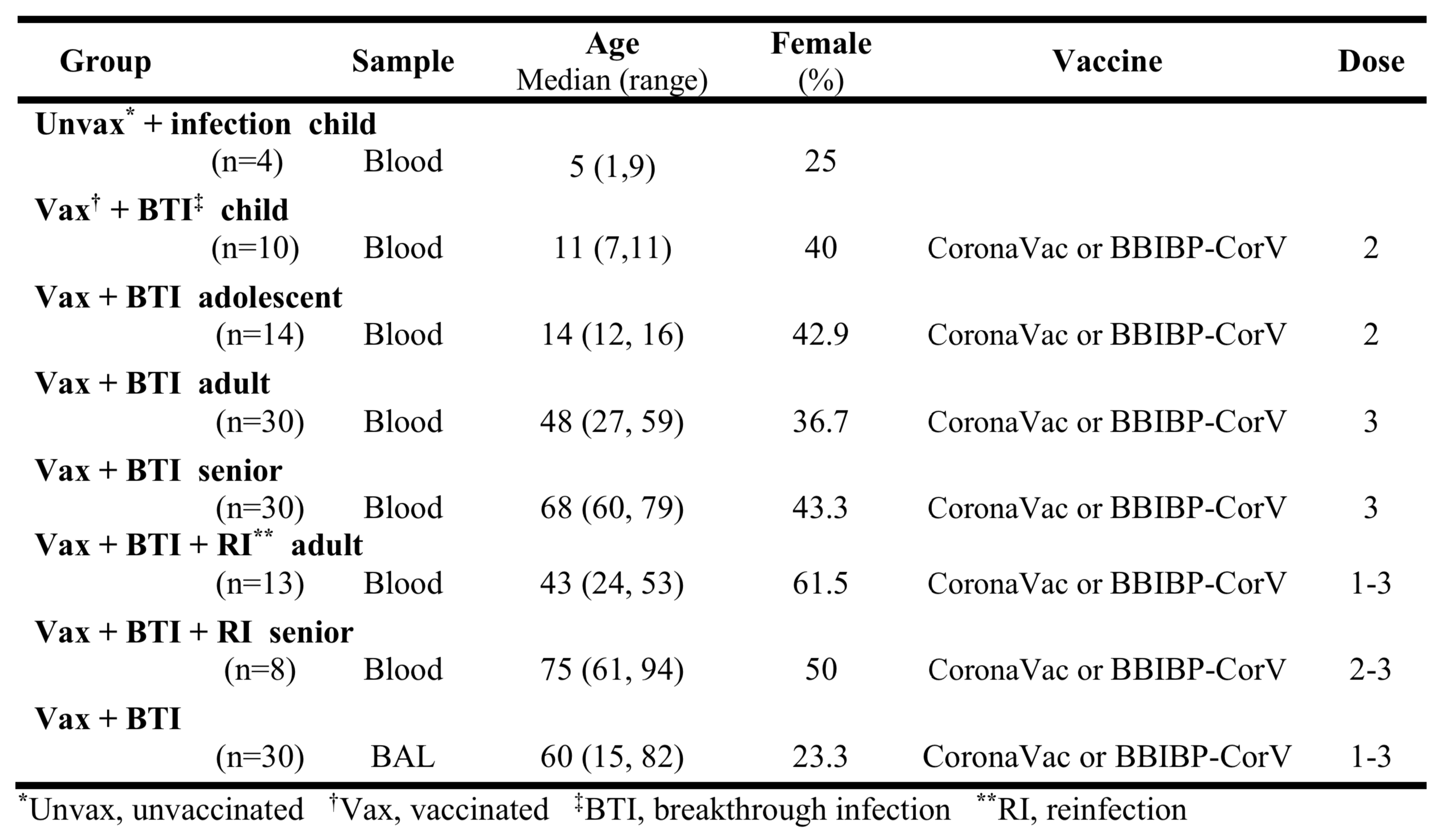
**
